# Supplementary material for: Stochastic optimal feedforward-feedback control determines timing and variability of arm movements with or without vision
Source: PLoS Comput Biol. 2021 Jun 11;17(6):e1009047. doi: 10.1371/journal.pcbi.1009047 (PMC8221793; doi:10.1371/journal.pcbi.1009047)
Supplement: Attachment — Submitted filename: response_letter.pdf [file pcbi.1009047.s002.pdf]

We thank the Reviewers and Editor very much for the thoughtful comments and recommendations. We believe that the present revision addresses all major points raised by the reviewers. Briefly:

- We now model movements without vision by considering larger observation noise. Hence, we consider that the state feedback is less reliable in this condition.
- We do not use a reference trajectory (to yield the stretch reflex and muscle elasticity as in our previous paper Berret & Jean, PloS Comp Biol, 2020), as this is not needed to model the results we focus on in this paper. The proposed model is now fully compatible with the minimal intervention principle of Todorov & Jordan (Nat Neuro, 2002).
- The velocity profiles are now more bell-shaped thus corresponding to the experimental observations. This results from an additional hand jerk term (Flash & Hogan, J Neurosc, 1985) and from decreasing the weight of the variance term in the feedforward cost function.
- We have improved the discussion of previous works (in particular the comparison with optimal feedback control theory and the cost of time theory).

We describe below how we have addressed the reviewers' comments point by point. The changes are indicated in blue in the revised manuscript.

Reviewer #1

The authors present a model of human upper limb control composed of a feed-forward specification of a control sequence determined by the minimization of variance and effort, coupled with a local, linearized state-feedback controller which penalises deviation from the expected trajectory under feedforward control. It is argued that the model captures natural features of human movement control, including implicit selection of reach time, and changes in movement variance dependent on the feedback condition. Other models typically specify movement time either arbitrarily or based on factors that do not result from the derivation of the control law.

Overall the paper is very well written, very clear and the modelling exercise appears to have been performed very cautiously. The paper complies with utmost scientific standards in terms of the accuracy of mathematical content, and clarity of the writing. However, I emphatically disagree with the relevance of the model to explain features of human sensorimotor control. The reason is that several modelling choices are simply wrong; the first being that reaching with or without vision is equated to open loop or closed loop control, which cannot be less compatible with the physiology. A second critical modelling choice is the use of a control model that consists in trajectory tracking, which is motivated for mathematical reasons to derive locally optimal feedback control laws around a nominal or expected trajectory, yet the possibility that the human brain uses trajectory tracking is debated, and evidence thereof is elusive if existing.

The first point is the fact that control without vision is not feedforward control. There is extended evidence that online feedback control uses limb afferent feedback to correct for error when the hand is unseen (Goodale, Pelisson and Prablanc, 1986, Nature, and following literature). Evidence for sophisticated feedback control based on limb afferent feedback has been extensively discussed and reviewed for instance in Scott, 2016 (TINS, 39 (8), 512-526). It is clear that vision contributes to reducing the variability of movement but the larger amount of variability observed when vision is absent should not be equated to feedforward control in a model of human reaching. Recent evidence of subtle effects of vision during reaching has been documented here (ito and Gomi, eLife, 9:e52380), and it is clear that changing visual information is not equivalent to open loop versus closed loop.

The second main contentious point is the hypothesis of trajectory tracking, which has been questioned in the work of Todorov and Jordan, 2002, in the task with different numbers of via-points. This led to

partially artificial debate about whether there is or not a trajectory tracking mechanism in the brain. In fact, it appears that the brain may or may not use a trajectory for movement control, it is a feature of task related instructions (Cluff and Scott, JNS 35(36):12465–12476). These two points are major limitations: that the paper uses open loop trajectories to model movements without vision is in complete disagreement with current evidence that 1- such open loop trajectory may not exist in the brain, and 2-movements without vision are not feedforward.

It does not take anything away from the quality of the modelling work but as a model of human sensorimotor control, the model assumptions are not valid. I find it difficult to make a clear recommendation, the study could be published as is if the authors acknowledge that it is a bio-inspired model of control but it is not a model of the human sensorimotor system. Should the authors wish to make it a candidate model in the field of neurophysiology, then they should profoundly revise the underlying assumptions and abandon the idea of feed forward control in the absence of vision.

Answer: We thank the reviewer for their deep analysis of our modeling work. Regarding the first point, while the open-loop assumption was to capture the main aspects of movements carried out without vision, we agree that it is preferable to consider the fact that movement without vision still have feedback from e.g. proprioception. Therefore, we have now simulated movements without vision using SFFC by assuming that the state estimation is less reliable without multisensory integration, i.e. with increased observation noise. This makes state feedback control less efficient and potentially prone to large errors if feedback gains are too large (increasing observation noise decreases feedback gains, as in the Ito & Gomi's paper cited above; hence assuming a large observation noise emphasizes the role of the feedforward motor command). Regarding the second point, we do not assume the tracking of a reference trajectory in the feedback cost anymore. Instead, we assume that task's achievement must be ensured by representing the goal of the task in the feedback cost. As such, the correction process simply tries to execute the task by minimizing the amount of control corrections, akin to the minimal intervention principle. We assume that the brain has access to a representation of the expected upcoming trajectory, which is important in our model to create a local linearized approximation of the task dynamics. It is not necessary to assume that the brain seeks to track any "desired" trajectory even if it has some knowledge of it. However, we would like to mention that the SFFC is a general framework which allows to implement the tracking of a reference trajectory, such as to model the effect of muscle elasticity and the stretch reflex as in Berret & Jean (Plos Comp Biol, 2020).

Major points

The motivation for the model is not clearly apparent, it is interesting that a model can make predictions about movement time, but it is also not a big problem to consider it is a free parameter, that is we can decide movement time (i.e. I want to move slower or faster). Is there a genuine need for a model in which movement time is not under volitional control? Is there an experimental manipulation other than the empirical observation that movement time vary, which would justify that this parameter is linked to biomechanics and noise factors?

Besides gaining fundamental knowledge, timing is critical for human-human or human-robot interaction tasks for instance. Very often, to adapt assistance adequately it will be critical to detect, not only the upcoming hand path or joint coordination but also the associated movement timing. Setting movement duration by hand cannot be satisfactory for a sensorimotor control model as it does not provide any explanation about how the brain would choose this duration. The many robust invariants documented in the literature, such as the increase of duration and speed with movement

extent, clearly demonstrate that the movement duration is not arbitrarily set by the brain. Our model makes some predictions which can be tested with a specific experiment. For instance, if additive motor noise increases, the preferred duration should decrease if all other parameters are kept unchanged. This may be addressed in future experiments. We added this in the Discussion (lines 432-434).

In general, the work by the groups of Shadmehr and Ahmed on the cost of time and temporal discounting of reward is acknowledged but under-represented. In fact, it is not clear that the explanation of movement time based on biomechanics only is required at all given that the temporal discounting of reward is a well-established phenomenon and that movement time is also at least partially under volitional control. Thus the ability to account for changes in movement time with biomechanical factors is likely valuable for specific conditions.

We highly appreciate the work by the groups of Shadmehr and Ahmed and in fact several studies of the first author (Berret et al., J Neurosc, 2016; Berret et al., Sci Rep, 2018) also investigated consequences of the cost of time hypothesis to determine the timing (or vigor) of movement. This is now mentioned more clearly in the Introduction (lines 56-60). However, the results of this paper suggest that it is also important to investigate the influence of biomechanical constraints on the cost of time and assess its critical role in the control of vigor. Note that while the SFFC model provides an alternative approach to explain the nominal timing of movement from neuromechanical principles, this does not exclude considering a cost of time, in particular to explain inter-individual differences and dependencies of movement timing on reward. From a modelling point of view, it is further possible to include a cost of time at the planning stage in the SFFC model, in the term l(m,u) of the feedforward cost. This is now mentioned in the Discussion at lines 414-434.

Specific points

Line 70: Stochastic optimal control considers state-feedback adjustments not necessarily linked to corrections for changes relative to a plan

It is true that the optimal controller of a general SOC problem depends on the (stochastic) state of the plant. However, if we assume a planning stage and a local approximation around the noise free trajectory, then we can work with state deviations and control deviations. We hypothesize here that the brain implements such a locally optimal stochastic controller for correcting task-related errors during execution. This is actually a common approach, which was used also in the iterative linear-quadratic-Gaussian framework of Li & Todorov (2007) for example.

Equation 3: Why not using the formalism of stochastic differential equations? The proposed equation is slightly abusive as dw almost certainly does not exist (i.e. it has infinite instantaneous variation with probability 1).

We initially used this formal notation to simplify the reading, mainly because we already denoted deviations using a “delta” symbol. However, we agree with the reviewer that the true meaning is in terms of stochastic differential equations. Hence, in this revised manuscript, we used more rigorous notations even though they may look slightly more complicated (see Figure 2, Results section, and equations therein).

Equation 9: It would be useful to unpack where the linearization comes from and show the derivation from the original system.

Thank you for this suggestion, which we implemented at lines 188-200.

Reviewer #2

This study presents a new model for the planning and control of reaching movements. This model combines open-loop control with feedback control and is able to explain both the timing and the variability of reaching movements with and without sensory feedback. To explain the movement duration, a cost function is used that includes both effort and variance resulting from constant and signal-dependent noise in the motor command. The predictions are compared to previously published data and to the data of a new experiment.

General

Modelling the planning and control of reaching movements has a long history in the field of computational motor control. Over the years, many different ingredients have been proposed to be essential, such as open-loop control, feedback control, motor noise, and cost functions that include factors such as smoothness, effort and variance. This model can be considered as a unique combination of earlier proposed ingredients. The approach is therefore not original, but that's not a problem. If this particular model has more predictive power than earlier models, this is scientific progress and an important result. To what extent that is the case for this study will be evaluated below.

The methodology seems generally sound and the paper is generally clearly written, although the writing can be improved as outlined below.

Major points

1. The model proposed here (SFFC) includes a feedforward and a feedback component. The feedforward-only version of the model is used to explain reaching without visual feedback, whereas the full version is used to explain reaching with visual feedback. It is however not correct to consider reaching without vision as reaching without any sensory feedback as there is always proprioceptive feedback. Reaching without any sensory feedback is only possible for deafferented patients. I therefore recommend to use data of such patients to test the predictions the feedforward-only version of the model, and to use the full model with increased sensory variance for reaching without visual feedback (but with proprioception).

We thank the reviewer for this thoughtful recommendation. We implemented the proposed changes, and now simulate movements without vision in healthy subjects using the complete SFFC model but with increased sensory variance. This does not change the main results but considers the proprioceptive feedback.

2. The relevance of a model is determined by the quality of its predictions. The better a model predicts actual behaviour, the more relevant the model. The authors demonstrate that their model can explain many characteristics of human reaching movements. However, Figure 6 shows that there is one important aspect of reaching movements that the model does not predict correctly: the velocity profile. Whereas actual velocity profiles are well-known to be bell shaped, with the peak velocity occurring slightly before half the movement duration (see also the authors' own data in Fig 6A,B), Figure 6C,D shows that the model predicts atypical velocity profiles with an initially slowly increasing speed, a peak velocity that is reached long after half the movement duration, followed by a rapid decrease to zero. Since these predicted velocity profiles differ strongly from actual velocity profiles, I cannot consider this as a model that predicts all important aspects of reach trajectories correctly. I recommend the authors to try to fix the model so that it predicts more realistic velocity profiles.

We agree that the velocity profiles did not correspond well to the patterns observed in experiments. We analyzed the origin of this phenomenon in our model. This asymmetry came from two reasons.

First, it is known that the minimum torque change can predict abnormally skewed velocity profiles for certain point-to-point horizontal movements (e.g. Klein Breteler et al., Motor Control, 2002). The minimum jerk model is considered to better capture the symmetric shape of velocity profiles for horizontal movements. Hence, we modified our feedforward cost to include a term minimizing the hand jerk. In turn, this shows that a large variety of costs can be included at the planning stage of SFFC. The idea of composite cost mixing kinematic and dynamic criteria fits with other papers that investigated the issue of trajectory formation via inverse optimal control techniques (e.g. Berret et al., Plos Comp Biol, 2011). Second, we found that the asymmetry was more pronounced with a larger weight of the variance cost (in the feedforward cost). Increasing this weight allows to decrease the movement duration and better fit the experimental values. Finally, in this revision we had to increase the magnitude of additive noise to match the larger variance exhibited by deafferented patients, which also contributes to decrease movement duration. Hence, the variance weight was set to smaller values to fit experimental durations, and this resulted in a more bell-shaped velocity profile.

3. Equation 12 suggests that the model corrects for both task-relevant and task-irrelevant deviations from the nominal plan. Is that correct? This is at odds with stochastic optimal feedback control which corrects only for task-relevant deviations, a phenomenon that has strong experimental support. I recommend modifying the model so that it does not correct for task-irrelevant deviations.

We initially implemented a feedback cost that corrects any deviations from the planned trajectory. We now consider that the feedback cost minimizes only task-relevant errors during the post-movement duration while it minimizes the amount of control corrections during the whole movement. Note that this is compatible with the minimal intervention principle (see lines 220-222).

4. The proposed model has a number of free parameters. The values of these parameters were determined on the basis of data of Wang et al. (2016), and then later used to make predictions for the new experiment. In principle, that's a clean approach. However, in L254ff it is mentioned that some of the parameters were changed to compare the model predictions to the data. Are these then still model predictions, or is this just a fit of the model? I found this part of the paper rather vague. There is nothing wrong with fitting your new model, but why then first pretend as if the model was first fit to an independent data set, so that proper predictions can be made?

We clarified the selection of free parameters (lines 491-558). We first used data of previous experiments (Wang et al. 2016 without vision, Gordon et al. 1995 with deafferented patients) to adjust the free noise parameters. The remaining parameters were selected to reproduce the movement timing reported in Wang et al. (2016). However, in the novel experiment presented in the paper participants had shorter movement durations and were much more variable, probably due to differences in the protocols (larger target's width, no arm's weight support etc.) and instructions given to the subjects. Thus, adjustments of the parameters were needed to quantitatively fit the current experimental data. If we had kept the same parameters, the qualitative predictions would agree with the data but errors (quantified in terms of root mean square deviations) would be larger (see Figure 6 and lines 302-340).

5. To simulate movement with visual feedback, it is assumed that the visual feedback provides information about the full state. However, the state includes joint angles, joint angles velocities and net joint torques (Eq 3). It's debatable whether vision provides information about joint angles (I don't look at my elbow during reaching), but it is certainly questionable that it provides information about joint angle velocities. Most problematic however, is the assumption that vision provides feedback about net joint torques - that's certainly not realistic.

Our model considers that when all sensory information is available the brain can accurately estimate the current state of the system from the fusion of the various sensory modalities, i.e. in the reaching arm movements considered in this paper mainly proprioception and vision. Therefore, we do not assume that vision per se provides information about net joint torques but combined with proprioceptive information and internal models of the limb kinematics and dynamics, the brain may be able to do that for such planar arm movements. This is also what has been hypothesized in the Cartesian point-mass model of Todorov (Neural Comp, 2005), which we refer to in the Introduction (Figure 1). For the sake of completeness, we also performed simulations by assuming that there is no sensory information about the net torque, and this did not significantly change the simulation results reported in the paper.

Minor points

The authors used ad hoc, non-standard acronyms for the various modelling approaches, such as DOC, SOC and SOOC. The logic of these acronyms is not always clear to me. For instance, SOC is used for stochastic optimal feedback control. Why not SOFC, as feedback is in my opinion the most crucial element of this approach?

We used SOC for stochastic optimal control because this is the term and abbreviation used by the control community from which it originates, where it is classically associated with a control that is a stochastic variable. Also, we thought here that SOFC may be confused with "stochastic optimal feedforward control". Hence, we decided to keep SOC as opposed to DOC in which everything is deterministic. Regarding SOOC, it is the acronym used in a previous paper of the first author, and is a sub-case of SOC, so we preferred to keep it unchanged. Finally, we used SFFC to stress the feedforward-feedback dichotomy of the approach in the new model.

L41-42: "SOC does not account for (…) the larger variance exhibited by movements without vision." That's not true, by increasing the level of variance in the sensory signals, this model predicts larger variance.

Thank you for noting this. We removed this part of the sentence, which was erroneous, and also modified Figure 1 to illustrate this fact (see new panel B in Fig. 1).

L56: greater -> longer

This was modified as suggested.

The Introduction is very long (it ends only after 201 of the 475 lines of text). In my opinion, L28-76 are the genuine introduction, whereas L77-201 could be absorbed into the Methods section.

It seems there has been a misunderstanding here as the Introduction was finishing at line 76 and was followed by a section on feedforward/feedback control. This section is now part of the Results section. We have also reorganized the manuscript and the Methods section, which gives the details about the numerical simulations and experiments, is now at the end of the paper (after the Discussion).

Fig 2, legend: I guess the corrective motor command can also be triggered by the consequences of noise in u.

The effects of internal noise are meant to be included in the term "perturbations". This is now clarified in the caption of Figure 2, which has been rewritten.

L110-111: “the originality of our proposal is to assume that the primary goal of motor planning is to build a feedforward motor command.” That does not sound very original. All the models not including feedback have this same goal.

We rephrased this paragraph to reflect more accurately the model’s specificity and how it extends previous models (see lines 91-109)

Eq 3: what is omega_t?

This is now explained at lines 130-133.

L147-149: “Note that hard constraints for the final mean or covariance of the state can also be added in this formulation. We did so for the final mean state to ensure that the arm exactly reaches the desired target on average…” I don’t see why the final mean state had to be set. It’s more natural to use a cost function such as mean squared error that allows for a biases that are accompanied by lower variance. For instance, the well-known undershoot of saccades has been explained this way (Chris Harris), whereas it allowed Liu & Todorov (2007) to explain incomplete corrections for target jumps.

We set the final mean state of the arm as a hard constraint in our model as this removes additional tuning parameters in the feedforward cost function, thereby simplifying the interplay between cost weights. However, the framework is more general and the target constraint could be implemented in the expected cost. Therefore, this should not be considered as a limitation of the model but a simplifying modeling choice, as is now explained at lines 160-175. Concerning endpoint biases like undershoots, the hard constraint can be removed and the goal of the task can be added in the feedforward cost. However, this should not be needed to explain incomplete corrections after a target jump as SFFC would handle this with the corrective feedback loop.

L175-176: Please back up the claim that delays in the sensory feedback are not critical.

Delays are obviously important in motor control in general thus we removed this sentence. Furthermore, we now simulate SFFC movements with a 50-ms delay in sensory feedback (see lines 552-558), which does not change the reaching movements considered in this paper much. Delays would have a critical importance in other tasks such as with instability, external perturbations, target jumps etc.

Methods: what time step was used in the model simulations, and do the model predictions depend on this time step?

The time step was set to dt=5 ms. This is now mentioned at lines 484-485. The predictions of the model are not sensitive to this choice of the time step if it is small enough, as was verified by testing with other time steps.

L264: than -> as

L279ff: “whose position was displayed on a monitor placed in from of the participant”. Whose position was displayed? Placed in FRONT of the participant? How was the monitor oriented?

These were modified as suggested. Thank you.

L295: “and reappeared at the end”. When exactly?

The cursor reappeared exactly 1 second after the end of the movement (lines 581-584). At the end of the movement, the participants were instructed to stand still until the cursor reappeared.

L300: So there were only 5 repetitions of each movement? That's way too few to obtain reliable estimates of the variability. At least several tens of repetitions are needed for this.

The experiment was designed mainly to compare the timing of movements with and without vision, and we did not plan to analyze the final variance. We agree that this is a small number of trials, however the increase of endpoint variability is large without vision and well noticeable for all distances, directions and participants. Also, it is well documented in the literature since Woodworth (1899). Therefore, we believe it is acceptable to have a rough estimate of the change of the overall endpoint variance in our task and use these data as a reference to compare with the model.

L307: what was the order of the filter?

We used a 5th order Butterworth low-pass filter. This is now indicated (at line 594).

L329: larger -> longer; L333: smaller -> shorter; L340: Fig 5C -> Fig 5D

Modified as suggested, thank you.

L347-400: The results of a large number of statistical tests are reported here. It is however unclear to me why all these tests were conducted. A statistical test is conducted to test a hypothesis. However, none of the tests addresses a hypothesis raised in this study. Instead, the focus of the study is on the model and the extent to which it can explain observed reaching movements. The comparison between predictions and data therefore deserves much more attention than the statistical tests on the data, but very little effort is put in this comparison. In summary, I recommend to remove all the statistical tests on the data and replace these by a critical comparison between the data and model predictions.

These statistical analyses are to test whether movements with and without vision differed in terms of timing, as our model does not predict drastic changes of movement with/out vision. For the sake of completeness, we also provided statistical analyses for the endpoint variability. We have now reduced the number of statistical results to focus on the main effects. Moreover, we added analyses that describe the root mean square deviation (RMSD) between the model and the actual data for the main variables under investigation (see Figure 6 and lines 302-340).

L409-410: "This learning will minimize feedback…" Unclear what is meant.

We have now clarified this sentence as follows: "This learning process will minimize the reliance on feedback corrections by gradually incorporating in the feedforward motor command any component that can be identified over trials." (lines 355-358).

L417-418: "One original aspect of SFFC is that the feedforward motor command considers uncertainty about the task dynamics…" This is not really original. Harris & Wolpert did this more than 20 years ago.

We agree with this comment and have thus replaced "original" by "important", and also better acknowledge the work of Harris & Wolpert. However, we can note a few differences between SFFC and their minimum variance model: the role of additive motor noise is important to shape the feedforward motor command and critical to determine movement duration (while it was not considered in the original work of Harris & Wolpert), and we clearly separate the variance and effort costs (while they were the same in their work).

L451: plan -> predict

This was modified as suggested.

Reviewer #3

In the current manuscript, the authors developed a new motor control model that explains the characteristics of timing (speed and duration) and variability of human reaching movements under the conditions of with and without visual feedback. The model which they call SFFC combines a feedforward control (SOOC in their model) and an online feedback control (SOC). The SOOC calculates an expected state trajectory and feedforward motor command in the planning phase and generates the movement in a feedforward manner. The critical point is that the motor command is optimized for the system stochasticity that includes not only signal-dependent noise, which is well known as a determinant of motor control, but also additive motor noise, which has not been given much importance in the field. As these two types of noise have opposing effects on the expected cost when the movement duration becomes longer, the optimal duration to minimize the cost is determined by the balance between the two. The motor planning by the SOOC can explain the timing characteristic that the movement duration increases as the target distance increases. Furthermore, it can account for an interesting observation reported by Wang et al (2016), which previous models fail to explain, that the duration of human preferred movements is greater than the minimum variance duration. On the other hand, the SOC corrects deviations online from the pre-planned state trajectory in a locally optimal manner. The SOC is assumed to be active only when visual feedback is available, which explains the variability characteristic that the variability is reduced in the presence of vision compared to when visual feedback is not available. The validity of their model was evaluated through comparison with human reaching data.

I think this model is original in that it explains the timing and variability characteristics without any ad hoc constraints, unlike previous models, but only by considering neuromechanical factors (noise properties, etc.). Also, this study tells us the importance of the motor planning considering additive motor noise in addition to signal-dependent noise. Thus, this study will bring new insight on the computational mechanisms underlying human motor control. However, I have three main concerns that will need to be addressed for this study to make a clear and reliable contribution to the literature.

1) First, the authors need more discussion on the currently dominating model, optimal feedback control model (OFC) (Todorov and Jordan, 2002). I agree that the OFC underestimates the importance of motor planning. However, the OFC is still a powerful model that can explain many features of human motor control. In order for the reader to better understand the difference in explanatory power between the OFC and the SFFC, the authors should more clearly mention (A) things the OFC can also explain with some additional assumptions, albeit ad hoc, and (B) things the OFC can explain but SFFC cannot if any.

(A): I acknowledge that the authors discuss carefully that the OFC can explain the timing characteristics by adding some assumptions as in previous studies. However, I feel the discussion on the difference between the variability with and without vision is insufficient. I wonder if the OFC could also explain an increased variability in the absence of vision when the weight of the observation noise increases due to only proprioceptive feedback available. If so, the authors should mention it.

This is correct, and we now include a new panel in Figure 1 (in the Introduction) to show that increasing the observation noise due to a lack of vision can explain the timing of movement without vision (just like SFFC does). This would also be explained by the cumulative effects of additive motor noise that are not properly correct due to inaccurate state feedback. However, it would not address the issue for visually guided movements since the same principle cannot be applied there. This is now better explained in the Introduction (at lines 38-51).

(B): As the SOC corrects only task-relevant movements (minimal intervention principle), variability (task-irrelevant) in the middle of the movement is greater than at the endpoint, which is consistent with human experimental data (Todorov and Jordan, 2002). In contrast, the SFFC corrects any deviation from a planned trajectory at every moment. Thus, I wonder if the SFFC could not explain the greater mid-movement variability. I would recommend that the authors add some discussion of this point.

The fact that SFFC corrects any deviation from a planned trajectory was just a specific instance of the model. We modified the model to make it compatible with the minimal intervention principle (see lines 210-232 and Eq. 14). By modifying the feedback cost function, the model now corrects deviations only when they affect the goal of the task, i.e. being on the target for the post-movement duration in this task (using the planned movement duration to define the post-movement period).

2) The second is regarding the interpretation of the experimental data. While the data for variability (Fig 7A,) clearly supports the validity of the model, the data for timing (Fig 7C, E) does not appear to be consistent with the model simulation. On line 456, the authors state that “the fact that movements with and without vision had similar timing in our data reaffirmed the importance of ….”. However, the ANOVA analysis in fact provides marginal significances with p=0.055 (duration) and p=0.052 (peak velocity) for the main effect of the visual condition although the model predicts no difference. Thus, I ask the author to reconsider the interpretation of the data and discuss what possible causes make the difference.

We now clearly mention that the p-values are close to statistical significance (e.g. lines 310-312 and line 330-332). We already described that the duration of movements with vision were longer than those without vision for the 24 cm distance. We may attribute this tendency of a slightly longer duration with vision to effects related to the integration of visual corrections at the end of the movement, to adjust more accurately the final cursor’s location. We added a few words on this point at lines 409-411 in the Discussion.

3) The third is the contribution of proprioception in the absence of vision. The SFFC assumes that in the absence of vision only the SOOC is active. However, it seems more plausible to assume that the proprioceptive feedback (with a large observation noise) is available and the SOC still works. Although the authors state that the SOC with a large observation noise amounts to use SOOC (lines 454-455), I feel that is a bit extreme. If the SFFC assumes the SOC with a large observation noise is active in the absence of vision, can it explain the increased variability in the absence of vision?

We used this rough assumption for movements without vision as it reproduced the drastic increase of endpoint variability, but we agree on the important role of proprioception also in movement without vision. To address this issue, we now simulate movements without vision by considering a large observation noise in the feedback correction as suggested by the reviewers.

Minor points

4) Figure1：The simulation result seems to change depending on the amount of additive noise and signal-dependent noise. When I run a simulation with Todorov’s model, I found the endpoint variance increases over the duration when the weight of additive noise is much greater than that of signal-dependent noise. The effect of increasing additive noise on endpoint variance is mentioned in Fig. 1B of Todorov (2005). Thus, the authors may modify the sentence in the caption, “The positional endpoint variance (gray trace) can also be seen to decrease and plateau to a value which mainly corresponds to that of visually-guided movements.”

We added a second panel to Figure 1 to depict the situation where a large observation noise is present, and to show that SOC with large observation noise can indeed lead to an increase of endpoint variance for long durations.

5) Line 46: Please remove “A”.

This was modified correspondingly.

6) Figure 4 AB: What do the black circles indicate? What are MD and VAR?

We modified the figure and better explain in the caption what is reported.

7) Figure 7: The presentation of experimental data requires a measure of variability.

As suggested, we added a measure of variability across participants in the figure (now Figure 6 in the revised version), where we used standard deviations as error bars.
