# Supplementary material for: Stochastic optimal feedforward-feedback control determines timing and variability of arm movements with or without vision
Source: PLoS Comput Biol. 2021 Jun 11;17(6):e1009047. doi: 10.1371/journal.pcbi.1009047 (PMC8221793; doi:10.1371/journal.pcbi.1009047)
Supplement: Attachment — Submitted filename: response_letter_rev2.pdf [file pcbi.1009047.s003.pdf]

We thank the Editors and Reviewers for their careful consideration of our manuscript, and describe below how we have addressed each of their comments. The changes are indicated in blue in the appended modified version.

Editor's comment

As you will see below review 1 (and indirectly the response to the previous version by reviewer 2) indicate that the equating of movement without vision and feedforward-only control is erroneous, and that the claim is still present in the manuscript. This needs to be clear in the paper that movement without vision can not be fully explained by feedforward mechanisms. This may require a minor or major rewrite, depending on whether you agree with this statement. Of course it might be possible instead to test experimentally (using deafferented patients, or with perturbation study) whether the feedforward aspect of the model alone can explain movement without vision, but that may be going further than your aim with this paper.

We agree that movements without vision cannot be fully explained by a feedforward-only mechanism (this is reflected in our model through the feedback contribution to the motor command). In this revision we have thus checked that no sentence wrongly suggests that the CNS uses pure feedforward control. In the following we address the specific comments of Reviewer #1 point by point.

Reviewer #1's comment

The authors have extensively revised the manuscript but I admit I feel that the idea of feedforward control corresponding to movements performed without vision is still there. Movements performed without vision are now associated with larger uncertainty but this modification falls a bit short of the more profound change that I believe was necessary.

To take a few examples, the abstract states the following: "[feedback] to correct task-related errors which are mostly noticeable when vision is available". This is wrong and misleading, there is extended evidence for non-visually mediated corrections for task-related errors. In the authors' summary, it is written "feedforward motor command before the movement starts, which can be observed for example in arm movements without vision […]". I do not feel it is useful to reiterate that movements without vision are not feedforward. This idea is pushed forward again later in the intro where it is written that: "SOC is designed to account for visually guided movements…" is also wrong. There is a double fallacy in this statement, first it ignores that feedback in SOC models needs not be visual, second it implies that when there is no vision SOC is probably not useful. This pushes the idea that movements without vision are feedforward, which I indicated in my first review does not stand one second in front of evidence that movement without vision are controlled in closed loop. The overall experimental work compares feedforward with movements performed without vision. The statement in the discussion that movement are largely based on feedforward control is again not supported.

The main message put forward is that changes in movement time can be captured by considering open loop minimization in the brain. This is interesting, but that does not imply that a feedforward controller is derived, much less executed. The experimental validation is not adequate and therefore the model is not supported. It is also conceivable that movement times are selected based on prior experience (including optimisation criteria, this is a contribution of the authors'), and that the implicit selection of movement time is used in feedback controllers with or without vision. To argue that movement performed without vision are similar to feedforward and establish that the selection of movement

time could be due to the optimization of a feedforward controller requires at least a task that involves perturbations. In support to their model the authors must demonstrate in a perturbation task that there is less or no feedback correction when there is no vision.

Much of the authors' reasoning to establish a similarity between no-vision and feedforward control is based on movement variability, but this reasoning is inconsistent with the work of Ito and Gomi (2020, eLife). The authors argumentation imply that corrections are reduced when vision is withheld, but there is another phenomenon, that is the gain of long-latency responses in this case in fact increased. Long-latency responses are not impedance control, and responses in the same time window increase without vision. This produced an increase in variance, but not due to less feedback correction, instead it was due to more vigorous corrections. This is a sufficient piece of evidence to reject the authors' model. That the model produces movement times that are consistent with human behaviour is interesting but the analogy stops there.

We thank the reviewer for looking at our manuscript and modifications carefully, and would like to address their comments in the following paragraphs.

First, we agree that obviously proprioceptive feedback is present in movements without online visual feedback: (For healthy subjects) movements are never under 'pure' feedforward control i.e. without sensory feedback. In our SFFC model, this corresponds to the feedback component of the motor command which is always present. As the reviewer pointed out, movements without online visual feedback are implemented in SFFC as increased uncertainty about hand state estimates (through the β parameter). This reduces the filter gain in the LQG layer and thus makes the controller "more feedforward", in the sense that the state estimate depends more strongly on the efferent copy and internal dynamical predictions. Even when the uncertainty about hand state estimate is large, sensory information is still used for online control. This reasoning is valid for both standard SOC models and the proposed SFFC model. We have checked and rephrased all related sentences to avoid any ambiguity and to clarify the role of proprioceptive feedback in movements without visual feedback.

Second, we agree that our data of unperturbed reaching movements do not allow to probe whether the strength of the feedback gains varies with different conditions such as with or without visual feedback. As indicated by the reviewer, this could be tested by a perturbation study such as carried out recently by Ito and Gomi (eLife, 2020), who found in particular that the feedback gains (i.e. in their case the EMG responses to mechanical perturbations) were reduced during wrist movements without online vision of the cursor. As Ito and Gomi further found that movement time and peak velocity did not vary with/out online visual feedback (like in our experimental results), they suggested that "to realize the predefined speed of movement we may need to assume that at least a part of the motor command is generated in a feedforward fashion (Bastian, 2006; Kawato, 1999; Sabes, 2000; Saijo and Gomi, 2010; Wolpert et al., 1998)". The rationale is that if the motor command was indeed based on pure state-feedback control with significantly reduced gains, movement speed should be reduced when there is no visual feedback. In the SFFC model, the unchanged kinematics regardless the magnitude of feedback gains is well explained by the feedforward component to the motor command that is not changed from the visual condition.

Third, if a mechanical perturbation was applied during the movement as in Ito and Gomi's study, the SFFC model would predict task-relevant motor corrections (that can be interpreted as a long-latency reflex response), characterized by the product between the state-feedback gain K(t) and estimated state deviations zhat_t (see lines 194-196). To illustrate the predictions of our model in a perturbation-based experiment, we simulated a perturbation applied to the elbow joint for a movement in the N-E direction (using the same parameters as in the main text of our paper and considering sensory

feedback with a 50ms delay). This arm movement mainly consists of an elbow extension about 10°, thus is close to the single-joint movement in Ito and Gomi's study. When an external extensor torque perturbation is applied to the elbow joint at about the middle of the movement (a 50ms long step of 0.25Nm), the SFFC model triggers a response that varies with/out visual feedback. The figure shows the motor correction in the elbow flexor torque compared to the baseline (unperturbed) torque. Noticeably, the corrective elbow flexor torque after perturbation is smaller without than with visual feedback, consistent with the lower EMG response found in Ito and Gomi's study. Since the feedback gains K(t) were maintained constant in our simulation, this difference stems from estimated limb state deviations (zhat_t) that are more strongly based on internal predictions in the absence of visual feedback due to the reduced filter gain with a larger observation noise.

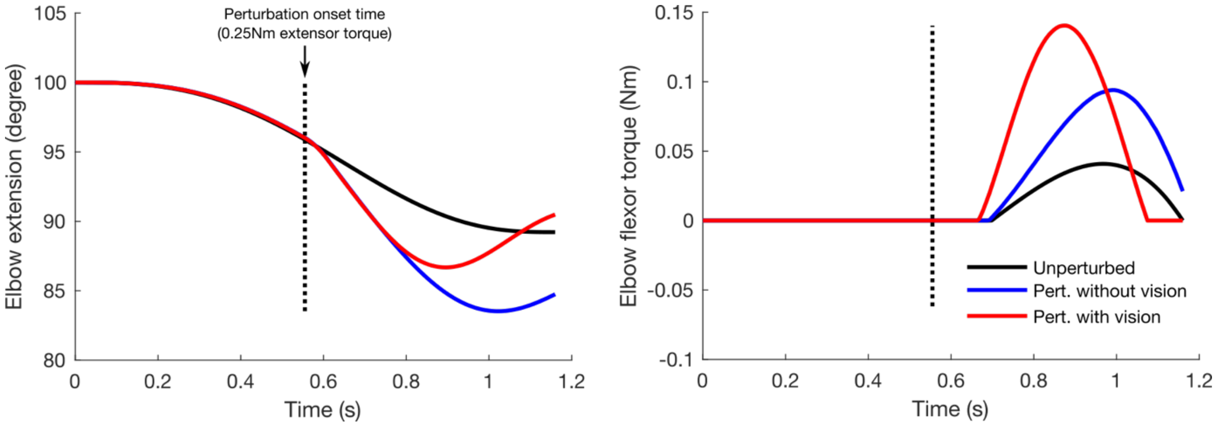

As mentioned by the reviewer, studies such as Ito and Gomi (eLife, 2020) or Izawa and Shadmehr (J Neuroscience, 2008) suggested that the feedback gains were reduced while others such as Mutha et al. (Brain Research, 2008) or Franklin et al. (J Neurophysiology, 2012) found increased feedback gains in response to various types of perturbations. The SFFC model could account for these task dependent gains: if the weight R in eq.14 increases (for instance when the task's goal is to quickly resist external perturbations), the state-feedback gain would be larger. Thus, the SFFC model can flexibly account for an increase or decrease of the feedback gains by adjusting R depending on the nature of the task and perturbation. This is mentioned in the paper at lines 214-216 and 525-528.

In view of all these results, we cannot agree with the reviewer's conclusion that there is sufficient piece of evidence to reject our model. On the contrary, our model is in very good agreement with Ito and Gomi's findings and interpretations. In particular, it allows to explain (via a nominal feedforward motor command) how motion kinematics can be well preserved even when measured feedback gains differ substantially.

The SFFC model unifies previous models related to optimal control theory of human movement and explains aspects that were not inherently explained by SOC, such as movement timing as we stressed here. Its predictions regarding the timing and variability of reaching movements with or without visual feedback are consistent with several experimental results. As is mentioned in the Discussion (at lines 396-398) and in Table 1, it is likely that SOC with a cost of time could also explain our experimental data of self-paced reaching movements. Further perturbation experiments (possibly with deafferented patients without vision) would indeed be necessary to better dissociate SOC and SFFC by clarifying whether the experimentally measured feedback gains can realistically drive the observed movement. Indeed, a key difference between SOC and SFFC is that the measured feedback gains are assumed to drive the movement in the former case (even without sensory feedback, using internal predictions, thus an open-loop mechanism) while they are assumed to serve only for the task-related corrective

behavior in response to internal or external perturbations in the latter case. However, we believe that testing this is out of the scope of the present study, and that it would require a dedicated study.

Other major points:

1. The limitation of SOC relative to changes in movement time is a straw-man, it simply follows from finite horizon formulation, so this model is not supposed to produce changes in movement time. The argumentation must be reworked throughout.

We believe that the time invariants observed in arm movements carried out in different conditions correspond to important characteristics of the motor control, which should thus be predicted by a computational model of arm reaching. The development of SFFC was initially motivated by the weakness of SOC in this respect. In finite-horizon formulation, duration is usually pre-specified (e.g. Liu and Todorov, J Neuroscience, 2007). In infinite-horizon formulation, a stopping criterion must be chosen (e.g. Qian et al., Neural Comp, 2013) and the velocity profiles are also very asymmetrical compared to the classical bell shape observed in self-paced reaching, which specifically corresponds to Fitts' law profiles. Our SFFC model was instead inspired by the fact that SOC with large observation noise can theoretically yield an optimal movement duration. Therefore, we modified the standard SOC framework to introduce a feedforward component in the motor command, which we used to predict the movement duration. As mentioned throughout the paper (lines 396-400 and Table 1), SOC with a cost of time could probably explain our experimental data, although carrying out the underlying simulations (e.g. with the traditional linear SOC model or with the iLQG algorithm of Li and Todorov, Int. J. Control, 2007) would be computationally challenging for the nonlinear arm dynamics we considered here. Our purpose and motivation to develop the SFFC model are now better clarified in the Introduction of the revised article, at lines 63-66.

2. Aspects of model presentation must be clarified: the procedure considering "fixed or free movement time" (just prior to Eqn. 4) was unclear and should be sketched out; pls sketch the argument why P(t) appears in the deterministic part of the SDE; it is unclear what manipulation was performed with the hard constraints that make the terminal penalty cost redundant or useless, what was the manipulation exactly?

Concerning the first point, the "fixed and free" was to mention that the simulations can be run by selecting a duration T or by letting the program automatically find it. We have now removed the "fixed or free" from this sentence as this point is presented in detail later in the text, at lines 159-166.

Concerning the second point on why P(t) appears in the "deterministic part of the SDE" (we assume that the reviewer refers to the propagation of the mean), we added details at lines 138-140.

Concerning the third point, there was no manipulation for the endpoint boundary condition as this simply corresponds to a modeling choice. If the final mean state is imposed, the arm will exactly reach the target on average. This "hard constraint" is set and does not result from a compromise in optimization process. Alternatively, a terminal penalty term can be used (as done in SOC) to specify where the system should converge to, however then the system never exactly reaches the target as it implements a compromise between fulfilling the goal task and expending effort. There thus is a weight tuning problem even for deterministic optimal control models using this approach. The SFFC model can use any of these two approaches. We do not claim that either approach is more valid, but used the former approach in our manuscript as it decreases the number of free parameters. This was explained at lines 152-157.

3. Page 7 middle: pls clarify, it is suggested that deafferented patients have both similar and higher level of variability, this section was unclear. The argument that variability in deafferented patients is

comparable in some conditions to control groups is not telling that they use the same control strategy, again a perturbation paradigm would clearly highlight differences between the two.

We have clarified the subsection "Comparison to previous data of reaching movements without vision". We agree that a perturbation paradigm would be critical to characterize the control in deafferented patients with respect to a normal population carrying out movements with/out visual feedback. However the paragraph at lines 231-249 merely referred to Gordon et al.'s (J Neurophysiology 1995) conclusion that "*the overall control strategy used by [deafferented] patients appeared the same as that used by controls [without vision]. Hand trajectories were approximately bell shaped, and movement extent was controlled by scaling a trajectory waveform in amplitude and time*". Gordon et al. also found that patients made larger errors than control subjects. Besides deficits in online feedback control, these patients also appear to have impaired motor planning. Therefore, a lack of proprioception may also affect internal models of limb dynamics such that the larger errors observed in deafferented subjects may be due the absence of online corrections or/and to inaccurate motor planning. The SFFC model assumes that a relatively accurate model of the dynamics has been learned and so it will not capture this impairment in deafferented patients. As a sidenote, the SFFC framework may be used in future work to describe how proprioception impairments affect feedback and feedforward control.

4. The correlation between timings of movements performed with or without vision is taken as evidence that they can share the same feedforward control command, an equally valid conclusion is that the consistent timing originated from the same state-feedback controller. This does not undermine the authors' efforts to show that movement times may be selected optimally, although there is no conclusive evidence that this selection is based on a feedforward controller.

While this result confirms a prediction of our model, we agree that it may also be predicted by other models such as SOC with the same state-feedback controller. We have modified the text at lines 382-384 correspondingly.

5. While the model parameters have likely been selected carefully it is necessary to perform a sensitivity analysis to assess the robustness of the model simulations against variations in parameter settings.

We have tested various sets of parameters and observed qualitative results clearly robust to such changes. For instance, a different set of parameters was used in the previous version of the paper, which exhibited similar qualitative results. Flexibility in the LQG component of the model can mainly affect variability, where different combinations of the parameters (ρ,β) can yield similar predictions for unperturbed reaching movements under consideration. For the SOOC component, changes in noise level would yield different durations without affecting the main observations, i.e. how the duration changes with respect to distance, direction etc. This is now mentioned in the paper at lines 486-493 and 518-528.

Reviewer #3

The new version submitted by the authors contains significant updates in their model as well as additional explanation/discussion on the model simulation and experimental data to answer to the points we raised in our first review. I appreciate the additional effort and think that the authors improved the quality of the paper which I consider now suitable for publication.

We thank the reviewer for their careful checking of the previous version and their comment.
